# New Insight into the Effects of Endogenous Protein and Lipids on the Enzymatic Digestion of Starch in Sorghum Flour

**DOI:** 10.3390/foods13050663

**Published:** 2024-02-22

**Authors:** Chen Chao, Song Liang, Zheyuan Zhang, Michael J. Gidley, Ye Liu, Shujun Wang

**Affiliations:** 1 State Key Laboratory of Food Nutrition and Safety, Tianjin University of Science & Technology, Tianjin 300457, China; chen.chao@tust.edu.cn (C.C.);; 2 School of Food Science and Engineering, Tianjin University of Science & Technology, Tianjin 300457, China; 3 Centre for Nutrition and Food Sciences, Queensland Alliance for Agriculture and Food Innovation, The University of Queensland, Brisbane, QLD 4072, Australia; 4 School of Food and Health, Beijing Technology and Business Technology, Beijing 100048, China

**Keywords:** sorghum, starch digestion, interaction, thermal processing, starch–lipid complex, starch–lipid–protein complex

## Abstract

The effects of endogenous lipids and protein in sorghum flour on starch digestion were studied following the depletion of lipids and/or protein and after the reconstitution of separated fractions. The removal of protein or lipids moderately increases the digestibility of starch in raw (uncooked) sorghum flour to values close to those for purified starch. Rapid Visco Analyzer data (as a model for the cooking process) show that cooked sorghum flours with lipids have a lower starch digestibility than those without lipids after RVA processing, due to the formation of starch–lipid complexes as evidenced by their higher final viscosity and larger enthalpy changes. Additionally, the formation of a starch–lipid–protein ternary complex was identified in cooked sorghum flour, rather than in a reconstituted ternary mixture, according to the unique cooling stage viscosity peak and a greater enthalpy of lipid complexes. After heating, the sorghum flour showed a lower digestibility than the depleted flours and the reconstituted flours. The results indicate that the natural organization of components in sorghum flour is an important factor in facilitating the interactions between starch, lipids, and protein during RVA processing and, in turn, reducing the starch digestion.

## 1. Introduction

Starch is the most abundant macronutrient in global diets and the main dietary energy source needed by humans. The rate and extent of starch digestion affect the postprandial blood glucose concentration and corresponding insulin release, which is important in relation to, e.g., the increasing incidences of type 2 diabetes and obesity [1,2,3]. The structural and physicochemical changes in starch during thermal processing (various kinds of household cooking and factory processing methods) and their effects on starch digestion have been widely studied [4,5]. However, starch normally exists with proteins and lipids in natural assemblies within grains and most cereal-based foods, and their interactions before and after food processing can significantly influence starch digestion [6,7,8,9]. Natural foods containing complex matrices are considered to be more nutritious and healthier than those assembled from isolated nutrients [10]. Due to the complex changes caused by thermal processing in food, the interaction mechanisms between starch, lipids, and proteins and their effects on the enzymatic digestion of starch in cereals is still far from understood. This has considerable practical relevance as well as theoretical interest for enhancing nutritional outcomes from cooked foods, such as in reducing the glycemic index and enhancing prebiotic properties.

Starch–lipid and starch–protein binary interactions and their effects on starch hydrolysis have been studied extensively in both model and cereal systems before and after boiling in water [11,12,13,14]. During this process, amylose in starch can form a single helical inclusion complex with lipids (termed the starch–lipid complex), reducing the rate of starch enzymic hydrolysis due to the hindrance of enzyme access to the substrate [15]. In contrast, the interaction between amylopectin and lipids is generally considered to be much weaker [16], although there is experimental evidence suggesting that long branch chains of amylopectin may also complex with lipids [17]. Both compact crystalline structures and the single helix conformation of amylose in complexes contribute to the decreased binding of amylolytic enzymes [18,19]. In contrast, endogenous protein matrices, which act as a physical barrier, can hinder and slow down enzyme diffusion toward starch substrates [20,21]. Some endogenous proteins are also reported to be inhibitors that reduce the activity of α-amylase [22]. The increased accessibility of α-amylase to starch after pre-digestion with protease has been reported previously [3,12,23,24]. However, there are inconsistencies when comparing the relative effects of lipids and proteins on starch digestion. In some cases, proteins were shown to play a more important role in affecting the physicochemical properties and digestion of starch than lipids [11,14,25,26,27], whereas other studies showed that the effects of lipids on the in vitro and in vivo starch digestibility of flour were more significant than those of proteins, especially after cooking in boiling water [13,28]. Moreover, most previous studies focused on the effects of the depletion of lipids and/or protein in flours before boiling on starch digestion in boiled flours, with little information on the role of the natural organization of the major components in raw (uncooked) sorghum flour in decreasing the starch digestibility of cooked flour.

Current evidence suggests that the current knowledge of the interactions between starch, lipids, and protein during food processing is not sufficient for understanding neither the properties of a formed structure nor the effects on starch digestibility when all three components are present [9,23,29]. The ternary interaction between starch, proteins, and lipids has been widely studied using a Rapid Visco Analyser (RVA). A combination of sorghum starch, whey proteins, and fatty acids exhibits a prominent viscosity peak during the RVA cooling stage, which has been attributed to the formation of amylose starch–lipid–protein ternary complexes (also referred to as the starch–lipid–protein ternary complex) [30,31]. Subsequent studies using techniques such as laser confocal micro-Raman (LCM-Raman) spectroscopy, Fourier transform infrared spectroscopy (FTIR), and X-ray diffraction (XRD) showed greater long- and short-range structural orders as well as a lower starch digestibility for the ternary complex than starch–lipid complexes [12,28,32]. Although the ternary interactions may also occur in cereal flour during cooking [28,29,33], little information is available regarding this ternary interaction’s effect on the enzymatic digestion of starch in cooked flour.

Sorghum (*Sorghum bicolor* (L.) Moench) is the third most important cereal behind wheat and barley in Australia, is a major crop worldwide, and has particularly attractive nutritional benefits compared with other cereals [34,35]. On the one hand, sorghum is an important ingredient for gluten-free products that are safe for celiac patients [36], particularly attractive because of its neutral flavor. On the other hand, sorghum, especially cooked sorghum, has a low starch digestibility, which has attracted attention as a possible route to reduce health risks associated with obesity [3,22]. The lower starch digestibility in both cooked and raw sorghum flour compared to that in other cereals has been attributed to the strong protein matrix (mainly composed of kafirins that are classified as prolamins) and starch–protein interactions that provide a physical restriction for amylolytic enzymes to access the starch granules [6].

However, the mechanisms by which the interactions between endogenous lipids, proteins, and starch in sorghum contribute to its low starch digestibility are still not well understood. In the present study, we systematically studied the effects of endogenous lipids and protein on starch digestion in uncooked and cooked sorghum flour by using depletion and reconstitution models. Through the characterization of pasting and the thermal properties of samples using an RVA and differential scanning calorimetry (DSC), a particular emphasis of the study was to unravel the mechanisms underlying the interactions between starch and endogenous lipids and protein, and their effects on starch digestion. Comparisons between depleted and reconstituted samples containing the same composition help to reveal the role of the natural structural organizations in sorghum in determining the interactions of components and their effects on starch digestibility. The knowledge gained is of interest for the development of sorghum-related foods with desired starch digestibility and glycemic responses.

## 2. Materials and Methods

### 2.1. Materials

Sorghum cultivars, TX430, were harvested from the University of Queensland farm (Gatton, QLD, Australia). A sorghum flour (SF) sample from whole grains of sorghum was prepared by milling through a 0.5 mm screen using a WonderMill pin mill (WonderMill Company, Pocatello, ID, USA) and was stored in a glass jar at room temperature. The cooked sorghum flour samples were obtained through Rapid visco analyzer (RVA) processing, which has been widely used as a suitable model for simulating thermal processes on a small scale under controlled conditions, as well as monitoring changes in the viscosity as the manufacturing process progresses [37]. Trypsin from porcine pancreas (Type IX-S, 13,000~20,000 BAEE units/mg protein), papain from Carica papaya (≥3 U/mg), and proteinase K from Tritirachium album (≥30 units/mg protein) used for protein removal were purchased from Sigma Chemical Co. (St. Louis, MO, USA). α-Amylase (PPA, A3176, type VI-B from porcine pancreas, 11 U/mg) and a total starch (AA/AMG) assay kit were purchased from Megazyme International Ireland Ltd. (Bray Co., Wicklow, Ireland). Other chemical reagents were all of analytical grade.

### 2.2. Sample Preparation

Sorghum starch (SS) and protein were isolated following the method of Zhang and Hamaker with modifications [22]. In total, 100 g of sorghum grains were steeped in 0.5% lactic acid and 0.2% SO_2_ solution (400 mL) for 36 h at 50 °C and were coarsely blended and then homogenized in distilled water for 10 min with a kitchen blender. The pH of the homogenate was adjusted to 8.5~9.0 using 0.5 M NaOH. After 0.5 h, the homogenate was passed through 100 mesh and 200 mesh wire sieves. The starch slurry was collected after sieving and was allowed to settle at 4 °C overnight. The supernatant was removed before resuspending the sedimented starch in distilled water and centrifuging at 2500× *g* for 15 min. The upper yellow layer of the pellet was collected, and the sediment starch was washed repeatedly via resuspension in water and centrifugation until the yellow layer was no longer evident. The remaining deposit was mixed with 10% toluene to completely remove the protein, and then 85% methanol was applied for 16 h with stirring at room temperature to remove the lipids. Isolated starch and protein were dried at 45 °C for one day.

The protein in sorghum flour was depleted via hydrolysis with a combination of proteases. A protease solution (300 mL) containing 0.5 g of trypsin, 0.5 g of papain, and 8 mg of proteinase K was prepared in 50 mM sodium Boric-Borax buffer (pH 8) and added to 30 g of sorghum flour. After overnight incubation at room temperature under constant magnetic stirring (200 rpm), the suspension was centrifuged at 3000× *g* for 10 min. The sediment was then washed three times with distilled water. The yellow protein layer of the residue was gently removed with the repeated addition of water and centrifugation until there was no visible yellow layer, and the protein-depleted sorghum flour (SF-P) was dried at 45 °C overnight.

The sorghum flour was defatted through diethyl ether extraction [38]. In total, 400 mL of petroleum ether was added into 20 g of sorghum flour with continuous stirring at room temperature for approximately 72 h. Filtration was carried out, and the solid residue was retained. These processes were repeated twice to obtain defatted sorghum flour (SF-L). After washing with anhydrous ethanol repeatedly until no petroleum ether residue was left, the sample was dried at 45 °C overnight.

### 2.3. Chemical Composition Analysis

The total starch content of all samples was measured using a Megazyme Total Starch kit according to a modified assay procedure (DMSO format) [39]. The protein content was measured using the Kjeldahl method and estimated by multiplying the determined nitrogen content by 6.25. The crude fat content was determined via Soxhlet extraction utilizing petroleum ether as the solvent (AACC, international, 2000) [40]. The moisture content was measured by drying the samples in an oven at 105 °C to a constant moisture content (AACC, international, 2000) [40].

### 2.4. In Vitro Starch Digestion

Samples containing 100 mg of starch (dry basis) were incubated in a phosphate-buffered saline (15 mL) solution with porcine α-amylase (1.6 units per mg of starch) at 37 °C with constant mixing (200 rpm). Aliquots (100 μL) were collected throughout a range of time points and added to a 900 μL Na_2_CO_3_ (0.3 M) solution to effectively stop further amylolysis reaction, followed by centrifugation at 2000× *g* for 10 min. The supernatant was used to determine the released maltose equivalent (reducing sugar) using the para-hydroxybenzoic acid hydrazide (PAHBAH) method reported previously [41]. Standards containing maltose concentrations of 4~500 μg/mL were also examined via PAHBAH assay.

Digestograms were fitted with the logarithm of slope (LOS) method [37], and rate coefficients were calculated as follows:

(1) $$\ln\left(\frac{dC}{dt}\right)=-kt+\ln\left(C_{\infty }k\right)$$

where ln(*dC*/*dt*) represents the logarithm of the slope, *k* (min^−1^) represents the starch digestion rate coefficient, and *C*_∞_ (%) is the estimated percentage of starch digested at the end of the reaction. This equation describes a linear relationship between the LOS and time of amylolysis, *t*, for a given rate coefficient.

### 2.5. Pasting Properties

The pasting properties of the SF, SF-L, SF-P, and SS samples were evaluated using a Rapid Viscosity Analyzer (RVA-4) (Newport Scientific, Warriewood, NSW 2102 Australia), which was also used as a simulated thermal processing model on a small scale under controlled conditions (Standard 1). In detail, samples containing 2 g of starch (dry basis) were mixed with distilled water to make a total weight of 28 g. The starch slurries were equilibrated at 50 °C for 2 min, heated at 8 °C/min to 95 °C, held at 95 °C for 3.5 min, cooled at 8 °C/min, and held at 50 °C for 3 min. The speed of the mixing paddle was 960 rpm for the first 10 s, and then 160 rpm for the remainder of the experiment. After measurements, the pastes were frozen in –80 °C immediately, freeze-dried, and ground into power using a mortar and pestle. The powders were passed through a 150 μm sieve and stored at 4 °C for structural analysis.

Starch, protein, and lipids were reconstituted in ratios found in raw sorghum flour and manually mixed by rotating the RVA paddle to obtain the starch–lipid (SS+L), starch–protein (SS+P), and starch–lipid–protein (SS+L+P) mixtures. All reconstituted samples were treated with the RVA protocol described above.

### 2.6. Swelling Power Analysis

The swelling power of starch granules was determined in excess boiling water according to the method of Zhang et al., [42]. The starch swelling power was calculated as the ratio of the weight of sedimented swollen granules to the dry weight of original starch.

### 2.7. Differential Scanning Calorimetry (DSC)

The thermal properties of samples were determined using a TA differential scanning calorimeter (DSC25, TA, New Castle, DE 19720, USA) equipped with thermal analysis data software (TRIOS 5.2). Samples (containing approximately 3 mg of starch, dry basis) were weighed accurately in an aluminum sample pan. Distilled water was added to obtain a ratio of starch (dry basis) to water of 1:3 (*w*/*w*). The pans were sealed and left to equilibrate overnight at room temperature. The sample and water mixture were held at 20 °C for 3 min, and then heated from 20 to 120 °C at a rate of 5 °C/min. An empty aluminum pan was used as the reference. Thermal transition parameters such as onset (T_o_), peak (T_p_), and end temperatures (T_c_) and the enthalpy of gelatinization (ΔH) were determined using the TRIOS 5.2 supplied with the instrument.

### 2.8. Statistical Analysis

All analyses were performed at least in triplicate, and the results were reported as the mean values and standard deviations. A one-way analysis of variance (ANOVA) followed by post-hoc Duncan’s multiple range test (*p* < 0.05) was conducted to determine the significant differences between mean values using the SPSS 19.0 Statistical Software Program (SPSS Inc., Chicago, IL, USA).

## 3. Results

### 3.1. Sample Characterization

The chemical compositions of the samples prepared in this study are shown in Table 1. The moisture content of all samples ranged from 6.7 to 11.3%. The total starch content of sorghum flour (64.4%) increased gradually after the removal of lipids (69.5%), protein (81.1%), and both (86.7%). Raw (uncooked) sorghum flour contained about 4.8% lipid, which was decreased to 0.2~0.5% after lipid extraction. Similarly, the protease treatment significantly reduced the protein content from 15.6% in sorghum flour to 3.5% for the SF-P sample, and to only 0.2% for isolated sorghum starch. There was a small decrease in protein (15.6%→14.4%) after the lipid extraction and removal of about half of the lipids (4.8%→2.3%) following protease treatment. These results suggest that the extraction treatments were not completely specific, which is consistent with lipid and protein co-localization and/or association in sorghum flour [43]. Nevertheless, the results show that defatting and deproteinization was generally effective, with treated products substantially depleted in the target component.

### 3.2. Effects of Endogenous Lipids and Proteins on Starch Digestibility in Sorghum Flour

**Figure 1:**
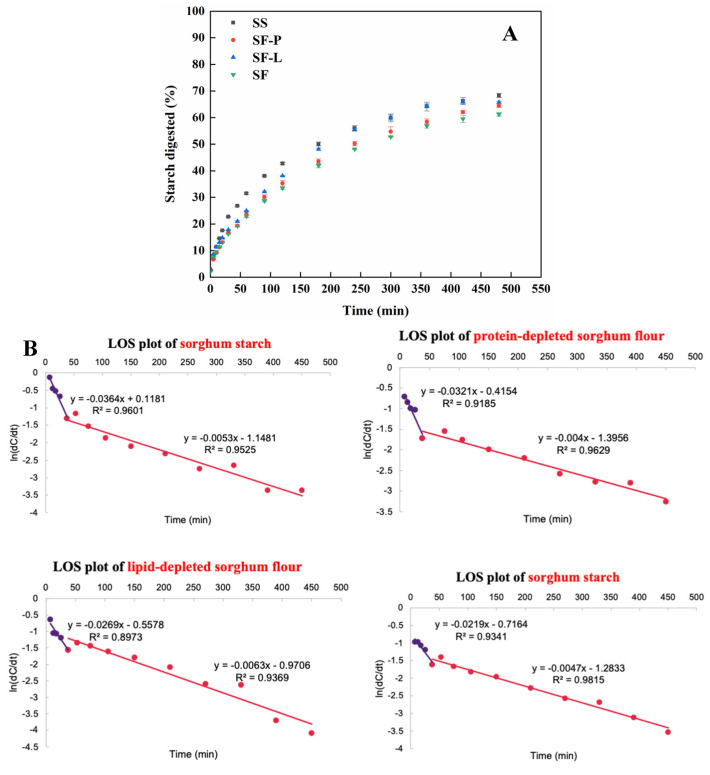

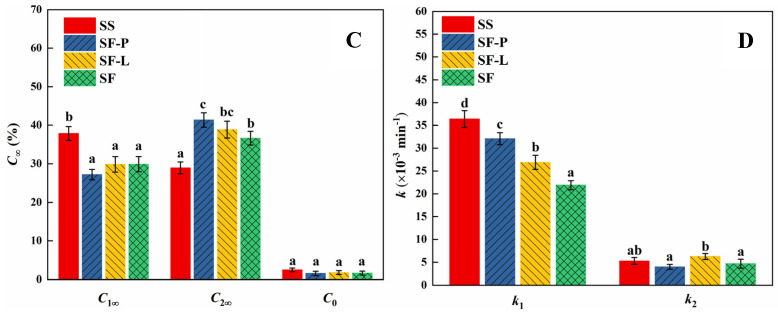
In vitro small intestine digestion properties of sorghum flour and its lipid/protein-depleted samples. (**A**) Digestion curves plotted as starch digested (%) as a function of time; (**B**) LOS plots of sorghum flour and its lipid/protein depleted samples; (**C**) Maximum starch amounts digested at infinite time for the digestible phases I and II (*C*_1∞_ and *C*_2∞_); (**D**) Starch digestion rate constants (*k* min^−1^) for the digestible phases I and II (*k*_1_ and *k*_2_). The part of the LOS plot describing *k*_1_ is shown in purple, and the part describing *k*_2_ is shown in red. Different letters on the top of the bars are significantly different at *p* < 0.05 (SF, sorghum flour; SF-L, sorghum flour after lipid extraction; SF-P, sorghum flour after protease treatment; SS, sorghum starch).

The in vitro small intestine digestion profile and first-order kinetic fitting parameters of (raw uncooked) sorghum flour (SF) and its lipid/protein-depleted samples (SF-P, SF-L, and SS) are shown in Figure 1. According to Figure 1A, starch in all samples digested slowly over the entire digestion period, which is consistent with previous studies [44,45]. SF-P and SF-L showed a moderately higher starch digestibility than SF, especially after 60 min of incubation, which indicated that the depletion of lipids or proteins facilitates the hydrolysis of starch in sorghum. The granular protein in raw sorghum flour is well known for decreasing starch digestibility by inhibiting α-amylase activity and as a physical barrier against starch–amylase binding [12,22,24]. The effects of lipids on starch digestibility can be mainly attributed to the presence of starch–lipid complexes in starch granules [9,28,46]. The magnitude of the increase in the starch digestibility of SS over SF approximates the sum of that of SF-P and SF-L.

The LOS plots of hydrolysis curves are shown in Figure 1B, exhibiting two first-order stages for all samples. The digested starch fraction in phase I (*C*_1∞_, ~40%) was higher than that in phase II (*C*_2∞_, ~30%) for sorghum starch, with the opposite seen for the SF, SF-P, and SF-L samples, indicating that the presence of lipids and/or protein leads to more slowly digestible starch (Figure 1B). The rate coefficient in phase I (*k*_1_) significantly increased from 21.9 min^−1^ for sorghum flour to 26.9, 32.1, and 36.4 min^−1^ for the SF-L, SF-P, and SS samples, respectively, (*p* < 0.05), while the *k*_2_ value was only slightly different between samples and much lower than *k*_1_ (Figure 1C).

### 3.3. Pasting Properties

**Figure 2:**
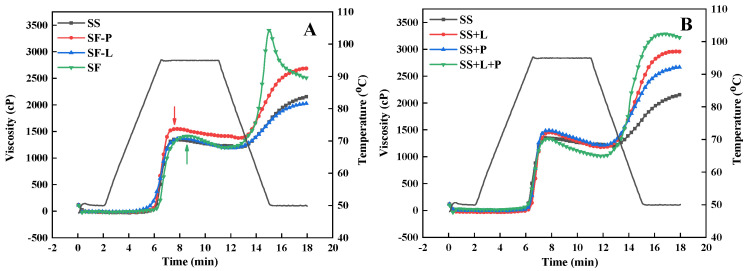
Rapid Visco Analyzer (RVA) profiles of raw (uncooked) lipid/protein-depleted (**A**) and reconstituted samples (**B**). SF, sorghum flour; SF-L, sorghum flour after lipid extraction; SF-P, sorghum flour after protease treatment; SS, sorghum starch; SS+L, sorghum starch reconstituted with lipids; SS+P, sorghum starch reconstituted with protein; SS+L+P, sorghum starch reconstituted with lipids and protein).

The RVA pasting profiles of raw protein/lipid-depleted and reconstituted samples are shown in Figure 2. A noticeable viscosity peak was observed in SF during the RVA cooling stage, while the depletion of proteins and/or lipids results in the disappearance of this viscosity peak (Figure 2A), which is consistent with a previous study [33]. These results can be attributed to the formation of starch–lipid–protein ternary complexes, which has been previously reported to result in a viscosity peak during the RVA cooling stage in starch–lipid–protein systems [30]. The SF-P sample showed a higher final viscosity than the SS and SF-L samples, which is due to the formation of starch–lipid complexes during the RVA cooling stage that accounts for the increase in viscosity [37,47]. Moreover, a significant delay in peak time (the time at which the maximum viscosity occurred during RVA heating) occurred in SF and SF-L compared to SS and SF-P, suggesting that sorghum protein hindered the swelling of starch granules during gelatinization. This is further supported by the results of the swelling power analysis (Table 1), showing that SF and SF-L have lower swelling capacities (8.3 and 10.0) than SS and SF-P (13.4 and 13.7, respectively). These results are consistent with results reported previously [21].

The pasting profiles of the reconstituted samples showed some differences to those of protein/lipid-depleted samples (Figure 2B). Of particular note is the lack of an obvious viscosity peak during the RVA cooling stage of SS+L+P compared with SF. Moreover, the addition of isolated sorghum protein to SS increased the starch final viscosity, whereas SF-L had a similar final viscosity to that of SS. This is likely due to the added protein being more readily capable of disulfide-crosslinking, which increases the final viscosity to a larger extent [48]. The addition of protein and lipids did not delay the viscosity peak time of starch gelatinization shown for protein/lipid-depleted samples, further suggesting that the different structural organizations of components between raw and reconstituted samples may influence their interactions during processing and pasting.

### 3.4. Thermal Properties

**Figure 3:**
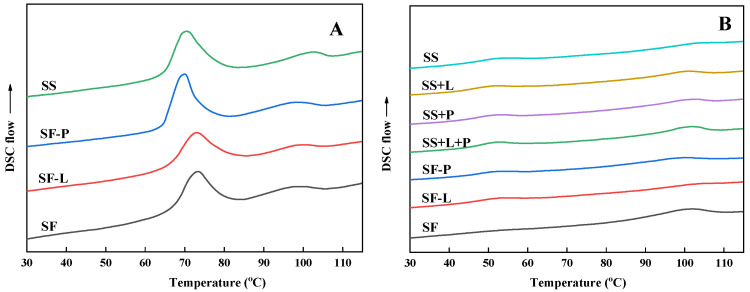
DSC curves of raw (uncooked) lipid/protein-depleted sorghum flours (**A**) and lipid/protein-depleted and reconstituted samples after RVA processing (**B**). (SF, sorghum flour; SF-L, sorghum flour after lipid extraction; SF-P, sorghum flour after protease treatment; SS, sorghum starch; SS+L, sorghum starch reconstituted with lipids; SS+P, sorghum starch reconstituted with protein; SS+L+P, sorghum starch reconstituted with lipids and protein).

The DSC thermograms of raw (uncooked) samples and freeze-dried pastes obtained after RVA processing are shown in Figure 3. Two endothermic transition peaks at ~71 °C and 98 °C were observed in flour samples without processing, corresponding to starch gelatinization and the melting of starch–lipid complexes, respectively (Figure 3A). After RVA processing, the endothermic peak for gelatinization disappeared for all samples. Two relatively broad peaks were observed at around 52 °C (except SF) and 100 °C (Figure 3B), which is indicative of the recrystallization of starch chains into double helices (retrogradation) and the formation of starch–lipid or starch–lipid–protein complexes during the RVA cooling stage and lyophilization, respectively [49,50].

The thermal transition temperatures (T_o_, T_p_, and T_c_) and enthalpy changes (ΔH) for samples before and after RVA processing are shown in Table 2 and Table 3, respectively. The peak temperature of the starch gelatinization for SF (73.2 °C), and to a lesser extent for SF-L (72.8 °C), was higher than those for the SS (70.1 °C) and SF-P (70.3 °C) samples, with the enthalpy change showing opposite trends (Table 2). The majority of sorghum grain proteins (termed kafirins) are encapsulated in protein bodies, whereas non-kafirins form a coating around the protein bodies that effectively bind them into a matrix that surrounds the starch granules in endosperm [51]. These observations further confirmed that sorghum grain proteins are linked by disulfide-bonded networks and that physical interactions occur between the starch and protein in planta [6,22,52]. These interactions restrict the swelling and gelatinization of starch granules, which is consistent with the longer RVA peak time and lower swelling powers of SF and SF-L compared with those of SS and SF-P. The enthalpy changes in the second endothermic peak, which mainly represents the amounts of starch–lipid complexes [37], decreased from 2.5 and 2.6 J/g for the SF and SF-P samples to 1.6 and 1.8 J/g for the SF-L and SS samples.

RVA processing increased the melting enthalpy of V-type complexes for all samples, especially for SF (from 2.5 to 6.9 J/g), indicating that the simulated cooking process promoted the formation of V-type complexes (Table 3). The ΔH_2_ values of SS (2.3 J/g) and SS-L (2.1 J/g) were less than half that of SF-P (4.8 J/g). However, the presence of protein and lipids significantly increased the enthalpy change to 6.9 J/g for SF, which was not observed in corresponding uncooked samples (Table 1: 2.6 J/g for SF-P and 2.5 J/g for SF), indicating that starch–lipid–protein complexes formed during RVA processing. It is known whether starch–lipid–protein complexes have larger enthalpy changes in V-type crystals than starch–lipid complexes [53]. Although significant increases in ΔH_2_ values were also observed in SS+L and SS+L+P compared with SS (reconstituted samples), their ΔH_2_ and T_p_ were lower than the corresponding depleted samples (SF-P and SF). These results indicate that a greater amount and more stable complexes formed in the natural sorghum system than in the reconstituted system. Accordingly, we demonstrated that the natural structural organization of components in sorghum are critical in facilitating the interaction between components during RVA processing, such as the accessibility of lipids and protein to starch. Moreover, the ΔH_1_ value of all samples decreased with ΔH_2_ increasing, and no enthalpy change associated with retrogradation was detected in the SF sample, which could be explained as being due to the formation of starch–lipid complexes inhibiting starch retrogradation [54].

### 3.5. Effects of Lipids and Proteins on Starch Digestibility after RVA Processing

**Figure 4:**
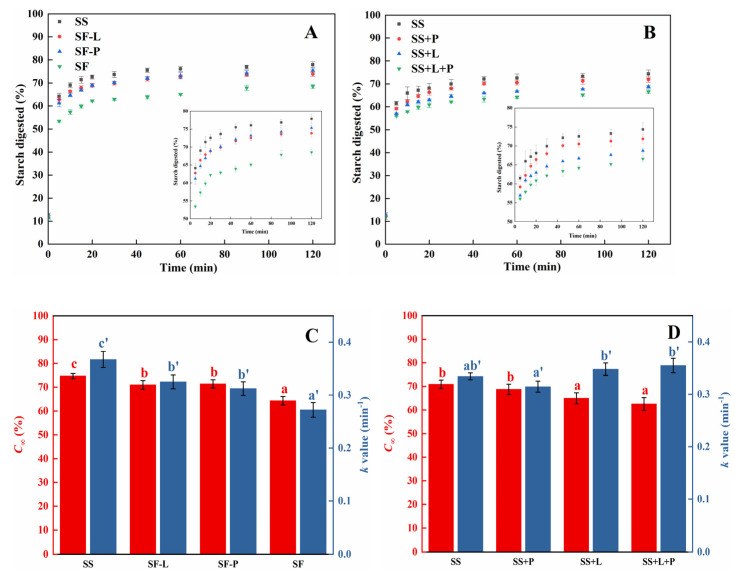
In vitro small intestine digestion curves plotted with the starch-digested ratio as a function of time and the first-order kinetic fitting parameters of the lipid/protein-depleted (**A**,**C**) and reconstituted samples (**B**,**D**) after RVA treatment. The red bars represent the maximum starch amounts digested at infinite time (*C*_∞_), and the blue bars represent the digestion rates (*k* value). Different letters on the top of the red or blue bars indicate values that are significantly different at *p* < 0.05 (SF, sorghum flour; SF-L, sorghum flour after lipid extraction; SF-P, sorghum flour after protease treatment; SS, sorghum starch; SS+L, sorghum starch reconstituted with lipids; SS+P, sorghum starch reconstituted with protein; SS+L+P, sorghum starch reconstituted with lipids and protein).

The in vitro α-amylase digestograms and the first-order kinetic fitting parameters of samples obtained after RVA processing are shown in Figure 4. Gelatinized samples presented different digestion patterns compared with the uncooked samples (Figure 4A). Most of the digestion occurred in the first 20 min (~70%), and then a plateau region was reached after a longer digestion time, which is in general agreement with previous research [13,14,22]. The sorghum flour displayed the lowest degree of starch hydrolysis over the whole digestion period (Figure 4A). The depletion of either lipids or proteins before RVA processing significantly increased the digestibility, with similar digestibility to gelatinized sorghum starch. This synergistic effect on starch digestibility occurred when lipids and proteins were both present in sorghum flour, which was also observed in a study on millet flour digestion [28]. For reconstituted samples, the gelatinized SS+L samples showed lower degrees of starch hydrolysis compared with SS+P, indicating that the effects of lipids on the in vitro digestibility of sorghum starch may be more significant than those of proteins after processing (Figure 4B).

A kinetic analysis showed that the depletion of proteins significantly increased the extent of the final digested starch fraction (*C*_∞_) and the digestion rate of gelatinized starch from 64.3% and 0.27 min^−1^ to 71.4% and 0.32 min^−1^, with the same effect also seen in the lipid-depleted sample (to 71.0% and 0.33 min^−1^) (Figure 4C). The depletion of both proteins and lipids results in a ~10% increase in *C*_∞_. However, the addition of protein to sorghum starch did not change the *C_∞_*, while the addition of lipids resulted in a 6% decrease in *C_∞_* (Figure 4D). These observations provide further evidence that the structural organization of macronutrients within the flour is important in determining the enzymatic digestion of starch after RVA processing.

## 4. Discussion

In this study, the effects of endogenous lipids and protein in sorghum flour on starch digestion were studied by removing most lipids and/or protein and reconstituting separated fractions. On the one hand, proteins and lipids were proposed to provide partial amylolysis resistance to different extents, related to interactions with starch and whether the flour undergoes cooking. On the other hand, the natural structural organization of the three macronutrients in sorghum flour favors ternary interactions during cooking, impacting starch digestion in a different way compared with that in reconstituted samples.

### 4.1. Proteins and Lipids Act Differently in Influencing Starch Digestibility in Uncooked Sorghum

Proteins and lipids in raw (uncooked) sorghum flour contribute to the innate resistance to enzymic digestion (Figure 1). In most cereals, protein bodies are embedded in a protein matrix that is associated with the surface of starch granules, which slows down the starch hydrolysis mainly by acting as a physical barrier hindering starch–amylase access/binding [6,21,23,24]. The effects of lipids on starch digestibility can be mainly attributed to the non-covalent interactions between amylose and lipids and the formation of V-type complexes. The helical conformation of amylose in complexes and their compact structure hinders the access and diffusion of enzymes to substrates [55]. Hence, the sorghum flour exhibits a lower, rapidly digestible starch fraction and higher, slowly digestible fraction compared with lipid- or protein-depleted samples. Sorghum proteins display a more significant effect on impeding the initial digestion rate than lipids, which might be due to the greater amounts of protein available for interaction with starch, as well as the limited amounts of amylose–lipid complexes naturally present in starch granules.

### 4.2. RVA Processing Facilitates Starch–Lipid Complex Formation and Reduces Starch Digestibility in Sorghum

Protein in sorghum flour can also contribute to the decreases in the degree and rate of starch hydrolysis after the crystalline structure of starch is disrupted during RVA processing [13,25,28]. It has been reported that the formation of a disulfide-bonded network in proteins can impede starch digestion, both by physically hindering amylase access to the substrate and restricting starch gelatinization [22,24]. Although no detectable ungelatinized starch was observed in this study, the short-range molecular order (residual double helical clusters and partially uncoiled double helices) was reported to exist in gelatinized starch, which can also affect starch digestibility [56,57]. Meanwhile, the reconstitution of starch and proteins before RVA processing, in which the interaction between starch and proteins was not likely to be the same as that in flour, had little effect on starch digestion, which is consistent with the unchanged peak time in RVA processing.

Endogenous lipids form more complexes with amylose after RVA processing as observed via DSC, which leads to a more significant decrease in starch digestibility in flour than that before cooking. Starch–lipid complexes are mostly formed during the setback period after starch gelatinization, resulting in an increase in the final viscosity [37], which was also observed in the RVA profiles of the SF-P and SS+L samples. The effects of lipids on the in vitro starch digestibility of processed millet were also found to be more significant than those of proteins [28]. Considering the significantly lower content of lipids (~5%) than proteins (~15%) in sorghum, it is reasonable to propose that lipids are more efficient in decreasing sorghum starch digestibility compared with protein, which provides important information for the optimization of food processing and the manipulation of the quality attributes of final food products.

### 4.3. Ternary Interactions during RVA Processing Enhance Resistance against Enzymatic Digestion

The ternary interaction between starch, lipids, and protein was first studied in model systems containing starch, whey protein, and fatty acids using an RVA [30,31]. A similar phenomenon was proposed for a natural sorghum flour system [33], with the formation of starch–lipid–protein ternary complexes and a viscosity peak during the RVA cooling stage [30,31,33,53]. Amylose, lipids, and protein can form ternary complexes involving a combination of non-covalent interactions such as hydrophobic forces and van der Waals forces between amylose and lipids, and hydrogen bonds and van der Waals forces between amylose and proteins such as β-lactoglobulin [58]. Ternary complexes are proposed to be more resistant than starch–lipid binary complexes to amylolysis, due to greater structural order and increased steric hindrance [50]. Nevertheless, the effects of starch–lipid–protein complex formation on starch digestibility has been studied only in model systems, while their effects on starch hydrolysis in natural flour systems or food products are still not clear.

Compared with sorghum starch and sorghum flour after lipid/protein depletion, a unique cooling stage viscosity peak and higher enthalpy change in V-type complexes were observed for SF samples, indicating that the ternary interaction occurs between starch, lipids, and protein in sorghum flour, which is consistent with previous studies [33]. However, these observations were not found in reconstituted ternary mixtures, although the final viscosity and melting enthalpy of complexes were higher than for starch–lipid mixtures. The depletion of lipids and/or proteins from sorghum flour before RVA processing shows a more significant influence on the extent and rate of enzymatic digestion than the reconstitution of lipids and/or proteins with starch. These results suggest that the natural structural arrangement facilitates starch–lipid–protein interactions and ternary complex formation in sorghum flour leading to the lower enzymatic susceptibility of ternary complexes compared with starch–lipid complexes, as reported previously. The protein bodies and lipids are close to starch granules in sorghum endosperm, which may allow the proteins and lipids to be more accessible to form ternary complexes, rather than tending toward self-assembly or disulfide-linking in reconstituted systems, thereby resulting in increased viscosity (Figure 3B). Hence, we conclude that the ternary interaction between starch, lipids, and proteins during processing should be considered when evaluating the effects on starch digestion of sorghum, and potentially other cereal-based foods.

## 5. Conclusions

In summary, our results show that endogenous lipids and protein can decrease the starch digestibility in sorghum flour due to their interactions with starch both before and during cooking. In raw (uncooked) sorghum flour, the presence of protein can decrease starch digestibility by enhancing the physical hindrance of amylase, while lipid contributes to the amylolysis resistance of sorghum flour in the form of natural amylose–lipid complexes. The effects of lipids on starch digestibility are enhanced after RVA processing due to the formation of more starch–lipid complexes with amylolysis resistance. Compared with the reconstituted components, starch, lipids, and protein that are naturally organized in sorghum flour can form ternary complexes during thermal processing, as evidenced by a viscosity peak during the cooling stage and a high V-type complex melting enthalpy. The formation of starch–lipid–protein complexes in sorghum flour leads to a significantly lower starch digestibility than for flours depleted of lipids or protein. It should be noted that the natural organization of components in sorghum flour is an important factor in facilitating the interactions between starch, lipids, and protein during RVA processing, and, in turn, reducing the starch digestion. The findings of this study contribute to the understanding of interactions between starch and other components in sorghum flour during cooking. This provides guidance on the preparation of sorghum-based foods with moderated starch digestibility and glycemic responses by controlling component interactions during cooking. Moreover, it will help as a guide when selecting from the varieties of sorghum (different ratios of starch, lipids, and proteins) for food preparation.

## Figures and Tables

**Table 1:** Basic chemical compositions of sorghum flours and corresponding lipid/protein-depleted samples.

Samples	Moisture (%)	Total Starch (%)	Lipid (%)	Protein (%)	Swelling Capacity (%)
SF	9.6 ± 0.1 b	64.4 ± 1.2 a	4.8 ± 0.2 d	15.6 ± 0.0 d	8.3 ± 0.37 a
SF-L	6.7 ± 0.1 a	69.5 ± 1.1 b	0.2 ± 0.1 a	14.4 ± 0.0 c	10.0 ± 0.21 b
SF-P	10.2 ± 0.2 c	81.1 ± 1.2 c	2.3 ± 0.0 c	3.5 ± 0.0 b	13.4 ± 0.19 c
SS	11.3 ± 0.1 d	86.7 ± 0.5 d	0.5 ± 0.1 b	0.2 ± 0.1 a	13.7 ± 0.33 c

Values are the mean ± SD. Means with different letters in a column differ significantly (*p* < 0.05). SF, sorghum flour; SF-L, sorghum flour after lipid extraction; SF-P, sorghum flour after protease treatment; SS, sorghum starch.

**Table 2:** Thermal transition parameters of raw (uncooked) sorghum flour and corresponding lipid/protein-depleted samples.

Samples	T_o1_ (°C)	T_p1_ (°C)	T_c1_ (°C)	ΔH_1_ (J/g)	T_o2_ (°C)	T_p2_ (°C)	T_c2_ (°C)	ΔH_2_ (J/g)
SF	65.4 ± 1.1 c	73.2 ± 0.1 d	80.0 ± 0.2 c	10.8 ± 0.1 a	88.1 ± 1.5 a	98.0 ± 0.6 a	105.3 ± 0.6 a	2.5 ± 0.3 b
SF-P	64.4 ± 0.1 a	69.9 ± 0.1 a	76.5 ± 0.2 a	13.6 ± 0.7 b	87.5 ± 1.7 a	98.2 ± 1.2 a	105.8 ± 0.7 ab	2.6 ± 0.2 b
SF-L	65.9 ± 0.1 b	72.8 ± 0.1 c	80.1 ± 0.1 c	11.3 ± 0.3 a	88.5 ± 0.5 a	98.5 ± 0.2 a	105.0 ± 0.2 a	1.6 ± 0.3 a
SS	64.5 ± 0.3 a	70.3 ± 0.0 b	77.7 ± 0.3 b	13.8 ± 0.3 b	92.9 ± 0.8 b	101.4 ± 0.6 b	106.6 ± 0.1 b	1.8 ± 0.4 ab

Values are the mean ± SD. Means with different letters in a column differ significantly (*p* < 0.05). SF, sorghum flour; SF-L, sorghum flour after lipid extraction; SF-P, sorghum flour after protease treatment; SS, sorghum starch.

**Table 3:** Thermal transition parameters of lipid/protein-depleted and reconstituted samples after RVA treatment.

	Melting of Retrograded Starch				Melting of V-Type Complex Crystals			
Samples	T_o1_ (°C)	T_p1_ (°C)	T_c1_ (°C)	ΔH_1_ (J/g)	T_o2_ (°C)	T_p2_ (°C)	T_c2_ (°C)	ΔH_2_ (J/g)
SF	ND	ND	ND	ND	86.5 ± 0.5 a	100.5 ± 0.2 a	108.6 ± 0.1 c	6.9 ± 0.0 e
SF-P	43.8 ± 0.6 a	52.2 ± 0.2 c	60.1 ± 0.7 ab	2.3 ± 0.1 ab	87.1 ± 1.4 a	100.3 ± 0.2 a	108.5 ± 0.5 c	4.8 ± 0.3 d
SF-L	43.2 ± 0.7 a	52.8 ± 0.2 d	60.8 ± 1.6 b	2.7 ± 0.3 c	91.6 ± 1.2 c	102.5 ± 0.7 b	109.8 ± 0.7 d	2.1 ± 0.4 a
SS	42.8 ± 0.1 a	51.0 ± 0.2 a	58.8 ± 0.7 a	3.0 ± 0.1 d	91.7 ± 0.3 c	102.8 ± 0.2 b	108.0 ± 0.5 c	2.3 ± 0.1 ab
SS+P	42.9 ± 0.1 a	51.2 ± 0.5 ab	59.1 ± 0.3 a	2.3 ± 0.1 ab	90.9 ± 0.2 bc	100.4 ± 0.2 a	106.3 ± 0.3 ab	2.7 ± 0.0 b
SS+L	43.5 ± 0.6 a	51.7 ± 0.6 bc	59.1 ± 0.3 a	2.5 ± 0.3 bc	91.3 ± 0.4 bc	100.7 ± 0.5 a	106.9 ± 0.4 b	3.7 ± 0.3 c
SS+L+P	43.4 ± 1.1 a	51.4 ± 0.3 ab	59.3 ± 0.5 a	2.0 ± 0.2 a	90.0 ± 0.9 b	100.5 ± 0.3 a	105.8 ± 0.4 a	4.3 ± 0.1 d

Values are the mean ± SD. Means with different letters in a column differ significantly (*p* < 0.05). SF, sorghum flour; SF-L, sorghum flour after lipid extraction; SF-P, sorghum flour after protease treatment; SS, sorghum starch; SS+L, sorghum starch reconstituted with lipids; SS+P, sorghum starch reconstituted with protein; SS+L+P, sorghum starch reconstituted with lipids and protein. ND means not detected.

## Data Availability

The original contributions presented in the study are included in the article, further inquiries can be directed to the corresponding authors.

## References

[1] Sun L., Miao M. (2020). Dietary polyphenols modulate starch digestion and glycaemic level: A review. Crit. Rev. Food Sci. Nutr..

[2] Meenu M., Xu B. (2019). A critical review on anti-diabetic and anti-obesity effects of dietary resistant starch. Crit. Rev. Food Sci. Nutr..

[3] Zhang G., Hamaker B.R. (2009). Slowly digestible starch: Concept, mechanism, and proposed extended glycemic index. Crit. Rev. Food Sci. Nutr..

[4] Esquivel-Fajardo E.A., Martinez-Ascencio E.U., Oseguera-Toledo M.E., Londoño-Restrepo S.M., Rodriguez-García M.E. (2022). Influence of physicochemical changes of the avocado starch throughout its pasting profile: Combined extraction. Carbohydr. Polym..

[5] Wang Y., Chen L., Yang T., Ma Y., McClements D.J., Ren F., Tian Y., Jin Z. (2021). A review of structural transformations and properties changes in starch during thermal processing of foods. Food Hydrocoll..

[6] Dhital S., Brennan C., Gidley M.J. (2019). Location and interactions of starches in planta: Effects on food and nutritional functionality. Trends Food Sci. Technol..

[7] Singh J., Dartois A., Kaur L. (2010). Starch digestibility in food matrix: A review. Trends Food Sci. Technol..

[8] Toutounji M.R., Farahnaky A., Santhakumar A.B., Oli P., Butardo V.M., Blanchard C.L. (2019). Intrinsic and extrinsic factors affecting rice starch digestibility. Trends Food Sci. Technol..

[9] Qi K., Yi X., Li C. (2022). Effects of endogenous macronutrients and processing conditions on starch digestibility in wheat bread. Carbohydr. Polym..

[10] Fardet A., Rock E. (2014). Toward a new philosophy of preventive nutrition: From a reductionist to a holistic paradigm to improve nutritional recommendations. Adv. Nutr..

[11] Ding Y., Cheng J., Lin Q., Wang Q., Wang J., Yu G. (2021). Effects of endogenous proteins and lipids on structural, thermal, rheological, and pasting properties and digestibility of adlay seed (*Coix lacryma-jobi* L.) starch. Food Hydrocoll..

[12] Lin L., Yang H., Chi C., Ma X. (2020). Effect of protein types on structure and digestibility of starch-protein-lipids complexes. LWT.

[13] Yang J., Gu Z., Zhu L., Cheng L., Li Z., Li C., Hong Y. (2019). Buckwheat digestibility affected by the chemical and structural features of its main components. Food Hydrocoll..

[14] Ye J., Hu X., Luo S., McClements D.J., Liang L., Liu C. (2018). Effect of endogenous proteins and lipids on starch digestibility in rice flour. Food Res. Int..

[15] Gutiérrez T.J., Tovar J. (2021). Update of the concept of type 5 resistant starch (RS5): Self-assembled starch V-type complexes. Trends Food Sci. Tech..

[16] Guraya H.S., Kadan R.S., Champagne E.T. (1997). Effect of rice starch-lipid complexes on in vitro digestibility, complexing index, and viscosity. Cereal Chem..

[17] Copeland L., Blazek J., Salman H., Tang M.C. (2009). Form and functionality of starch. Food Hydrocoll..

[18] Ai Y., Hasjim J., Jane J.-L. (2013). Effects of lipids on enzymatic hydrolysis and physical properties of starch. Carbohydr. Polym..

[19] Zhang B., Huang Q., Luo F.X., Fu X. (2012). Structural characterizations and digestibility of debranched high-amylose maize starch complexed with lauric acid. Food Hydrocoll..

[20] Do D.T., Singh J., Johnson S., Singh H. (2022). Probing the Double-Layered Cotyledon Cell Structure of Navy Beans: Barrier Effect of the Protein Matrix on In Vitro Starch Digestion. Nutrients.

[21] Wang Z., Fan M., Hannachi K., Li Y., Qian H., Wang L. (2023). Impact of red kidney bean protein on starch digestion and exploring its underlying mechanism. Int. J. Biol. Macromol..

[22] Zhang G., Hamaker B.R. (1998). Low α-amylase starch digestibility of cooked sorghum flours and the effect of protein. Cereal Chem..

[23] Wu J., Warren F.J. (2023). The impact of the soluble protein fraction and kernel hardness on wheat flour starch digestibility. Food Chem..

[24] Xu H., Zhou J., Yu J., Wang S., Wang S. (2021). Mechanisms underlying the effect of gluten and its hydrolysates on in vitro enzymatic digestibility of wheat starch. Food Hydrocoll..

[25] Hou D., Zhao Q., Yousaf L., Xue Y., Shen Q. (2020). In vitro starch digestibility and estimated glycemic index of mung bean (*Vigna radiata* L.) as affected by endogenous proteins and lipids, and exogenous heat-processing methods. Plant Food Hum. Nutr..

[26] Yang Y., Jiao A., Zhao S., Liu Q., Fu X., Jin Z. (2021). Effect of removal of endogenous non-starch components on the structural, physicochemical properties, and in vitro digestibility of highland barley starch. Food Hydrocoll..

[27] Yang Y., Jiao A., Liu Q., Ren X., Zhu K., Jin Z. (2022). The effects of removing endogenous proteins, β-glucan and lipids on the surface microstructure, water migration and glucose diffusion in vitro of starch in highland barley flour. Food Hydrocoll..

[28] Annor G.A., Marcone M., Bertoft E., Seetharaman K. (2013). In vitro starch digestibility and expected glycemic index of kodo millet (*Paspalum scrobiculatum*) as affected by starch–protein–lipid interactions. Cereal Chem..

[29] Parada J., Santos J.L. (2016). Interactions between starch, lipids, and proteins in foods: Microstructure control for glycemic response modulation. Crit. Rev. Food Sci. Nutr..

[30] Zhang G., Hamaker B.R. (2003). A three components interaction among starch, protein, and free fatty acids revealed by pasting profiles. J. Agric. Food Chem..

[31] Zhang G., Maladen M.D., Hamaker B.R. (2003). Detection of a novel three component complex consisting of starch, protein, and free fatty acids. J. Agric. Food Chem..

[32] Kang X., Sui J., Zhang X., Wei G., Wang B., Liu P., Qiu L., El-Banna H.A., Cui B., Abd El-Aty A. (2022). The impact of gliadin and glutenin on the formation and structure of starch-lipid complexes. Food Chem..

[33] Zhang G., Hamaker B.R. (2005). Sorghum (*Sorghum bicolor* L. Moench) flour pasting properties influenced by free fatty acids and protein. Cereal Chem..

[34] Rooney L., Pflugfelder R. (1986). Factors affecting starch digestibility with special emphasis on sorghum and corn. J. Anim. Sci..

[35] Benmoussa M., Suhendra B., Aboubacar A., Hamaker B.R. (2006). Distinctive sorghum starch granule morphologies appear to improve raw starch digestibility. Starch-Starke.

[36] Ciacci C., Maiuri L., Caporaso N., Bucci C., Del Giudice L., Massardo D.R., Paola P., Natale D.F., Bean S.R., Brian I. (2007). Celiac disease: In vitro and in vivo safety and palatability of wheat-free sorghum food products. Clin. Nutr..

[37] Balet S., Guelpa A., Fox G., Manley M. (2019). Rapid Visco Analyser (RVA) as a tool for measuring starch-related physiochemical properties in cereals: A review. Food Anal. Methods.

[38] Concepcion J.C.T., Ouk S., Riedel A., Calingacion M., Zhao D., Ouk M., Garson M.J., Fitzgerald M.A. (2018). Quality evaluation, fatty acid analysis and untargeted profiling of volatiles in Cambodian rice. Food Chem..

[39] Edwards C.H., Warren F.J., Milligan P.J., Butterworth P.J., Ellis P.R. (2014). A novel method for classifying starch digestion by modelling the amylolysis of plant foods using first-order enzyme kinetic principles. Food Funct..

[40] Committee AAoCCAM (2000). Approved Methods of the American Association of Cereal Chemists.

[41] Li H., Dhital S., Gidley M.J., Gilbert R.G. (2019). A more general approach to fitting digestion kinetics of starch in food. Carbohydr. Polym..

[42] Zhang X., Guo D., Xue J., Yanniotis S., Mandala I. (2017). The effect of salt concentration on swelling power, rheological properties and saltiness perception of waxy, normal and high amylose maize starch. Food Funct..

[43] Osagie A.U. (1987). Total lipids of sorghum grain. J. Agric. Food Chem..

[44] Wang Y., Chao C., Huang H., Wang S., Wang S., Wang S., Copeland L. (2019). Revisiting mechanisms underlying digestion of starches. J. Agric. Food Chem..

[45] Guo P., Yu J., Copeland L., Wang S., Wang S. (2018). Mechanisms of starch gelatinization during heating of wheat flour and its effect on in vitro starch digestibility. Food Hydrocoll..

[46] Panyoo A.E., Emmambux M.N. (2017). Amylose–lipid complex production and potential health benefits: A mini-review. Starch-Starke.

[47] Tang M.C., Copeland L. (2007). Analysis of complexes between lipids and wheat starch. Carbohydr. Polym..

[48] Verbeek C.J., Van Den Berg L.E. (2010). Extrusion processing and properties of protein-based thermoplastics. Macromol. Mater. Eng..

[49] Dobosz A., Sikora M., Krystyjan M., Tomasik P., Lach R., Borczak B., Berski W., Lukasiewicz M. (2019). Short-and long-term retrogradation of potato starches with varying amylose content. J. Sci. Food Agr..

[50] Wang S., Chao C., Cai J., Niu B., Copeland L., Wang S. (2020). Starch–lipid and starch–lipid–protein complexes: A comprehensive review. Compr. Rev. Food Sci. Food Saf..

[51] De Mesa-Stonestreet N.J., Alavi S., Bean S.R. (2010). Sorghum proteins: The concentration, isolation, modification, and food applications of kafirins. J. Food Sci..

[52] Hamaker B.R., Bugusu B.A. Overview: Sorghum proteins and food quality. Proceedings of the Workshop on the Proteins of Sorghum and Millets: Enhancing Nutritional and Functional Properties for Africa [CD].

[53] Wang S., Zheng M., Yu J., Wang S., Copeland L. (2017). Insights into the formation and structures of starch–protein–lipid complexes. J. Agric. Food Chem..

[54] Mariscal-Moreno R.M., Figueroa-Cárdenas JD D., Santiago-Ramos D., Rayas-Duarte P. (2019). Amylose lipid complexes formation as an alternative to reduce amylopectin retrogradation and staling of stored tortillas. Int. J. Food Sci. Technol..

[55] Tan L., Kong L. (2020). Starch-guest inclusion complexes: Formation, structure, and enzymatic digestion. Crit. Rev. Food Sci. Nutr..

[56] Chi C., Li X., Huang S., Chen L., Zhang Y., Li L., Miao S. (2021). Basic principles in starch multi-scale structuration to mitigate digestibility: A review. Trends Food Sci. Tech..

[57] Santamaria M., Garzon R., Moreira R., Rosell C.M. (2021). Estimation of viscosity and hydrolysis kinetics of corn starch gels based on microstructural features using a simplified model. Carbohydr. Polym..

[58] Wang C., Chao C., Yu J., Copeland L., Huang Y., Wang S. (2022). Mechanisms underlying the formation of Amylose–Lauric Acid− β-Lactoglobulin complexes: Experimental and molecular dynamics studies. J. Agric. Food Chem..

